# Impact of surgical timing on postoperative quality of life in acute cholecystitis: a comparative analysis of early, intermediate, and delayed laparoscopic cholecystectomy

**DOI:** 10.1007/s00464-025-11620-9

**Published:** 2025-02-25

**Authors:** Azad Gazi Şahin, Erman Alçı

**Affiliations:** Department of General Surgery, School of Medicine, Balikesir University Hospital, 10463 Balikesir, Turkey

**Keywords:** Acute cholecystitis, Laparoscopic cholecystectomy, Surgical timing, Postoperative quality of life, Early intervention, Gastrointestinal quality of life ındex (GIQLI)

## Abstract

**Background:**

Acute cholecystitis, primarily caused by gallstones, is a serious condition that may lead to severe complications. The optimal timing of surgery for acute cholecystitis is still under debate. Early cholecystectomy is generally preferred to prevent complications and improve postoperative outcomes. This study aimed to evaluate the impact of early, intermediate, and delayed laparoscopic cholecystectomy on postoperative quality of life (QoL) in patients with acute cholecystitis.

**Methods:**

This retrospective study included 201 patients who underwent laparoscopic cholecystectomy for acute cholecystitis between May 2019 and February 2023. Patients were categorized into three groups based on the timing of surgery: early (within one week), intermediate (1–6 weeks), and delayed (after six weeks). The Gastrointestinal Quality of Life Index (GIQLI) was used to evaluate QoL six months postoperatively. Data on patient demographics, surgery timing, and cholecystitis severity (based on the Tokyo Guidelines) were analyzed using univariate and multivariate regression models.

**Results:**

The mean age of patients was 56.0 ± 14.9 years, and 65.7% were female. Early cholecystectomy was performed in 30.8% of cases, intermediate in 16.9%, and delayed in 52.2%. The median GIQLI score was 116. Patients who underwent early surgery had significantly higher GIQLI scores compared to those in the intermediate group (p < 0.001). No significant difference was observed between early and delayed surgery (p = 0.199). Multivariate analysis showed that intermediate surgery negatively affected QoL (p < 0.001), while cholecystitis severity was also a significant factor (p = 0.006).

**Conclusions:**

Early laparoscopic cholecystectomy significantly improves postoperative QoL compared to intermediate surgery. Delayed surgery provides similar QoL outcomes to early surgery. However, intermediate cholecystectomy may lead to poorer QoL due to heightened surgical complexity and increased complications. Early intervention should be prioritized to optimize patient outcomes.

**Supplementary Information:**

The online version contains supplementary material available at 10.1007/s00464-025-11620-9.

Acute cholecystitis is an acute inflammation of the gallbladder. It is a significant condition that can range from mild to severe and may become life-threatening due to its complications [1]. The clinical manifestations of acute cholecystitis typically include right upper quadrant pain, fever, and leukocytosis. However, pathological changes in the gallbladder may progress to a spectrum of conditions ranging from mild inflammation to empyema and gangrene [2]. In 90% of cases, gallbladder stones are the primary etiologic factor in acute cholecystitis, a condition referred to as calculous cholecystitis [1]. Acalculous cholecystitis, however, may be caused by factors such as postoperative complications, direct chemical injury, infections (microbial, protozoal, or parasitic), collagen tissue diseases, allergic reactions, and, in rare cases, tumors [3].

The main treatment for acute cholecystitis is cholecystectomy [1]. There has been considerable debate, however, regarding the optimal timing of surgical intervention. According to the Tokyo Guidelines 2018: Flowchart for the Management of Acute Cholecystitis, surgery performed within the first 72 h to one week after symptom onset is defined as early, while surgery performed at least six weeks later is considered delayed cholecystectomy. According to these guidelines, early surgery is recommended as the ideal acute cholecystectomy, regardless of the time of onset, if the patient is thought to be able to tolerate surgery [4]. The reason for delayed surgery is that inflammatory tissue is more susceptible to surgical complications, and more frequently exposed to adverse effects of surgery. Therefore, inflammation is typically reduced by delaying the surgery for at least six weeks [5]. However, studies have shown that early cholecystectomy is associated with a decrease in the incidence of wound infection and shorter hospitalization time and a significant improvement in the quality of life (QoL). Early surgery eliminates gallstone-related recurrent disease, which occurs in 2.5% to 22% of conservatively treated patients [3, 6]. In the period between early and delayed surgery, cholecystectomy is performed to treat complications such as empyema, gangrene, and perforation [5].

Although there are many studies on mortality, morbidity, and postoperative comfort of life in patients admitted to medical centers with the indication of acute cholecystitis, the literature on intermediate cholecystectomy is limited. The aim of this study is to assess the QoL of patients who presented to our center with acute cholecystitis and underwent early, intermediate, or delayed laparoscopic cholecystectomy in the postoperative period.

## Materials and methods

This is an observational, retrospective cohort study in which a total of 201 patients who underwent surgery for acute cholecystitis between May 2019 and February 2023 in the General Surgery clinics of Balikesir University Hospital were examined. The study was approved by Balikesir University Clinical Research Ethics Committee (Date: 22.11.2023; No: 2023/178). Informed consent was obtained from all patients before the operation. Patients who underwent early, intermediate, or delayed surgery for acute cholecystitis were included in the study, and laparoscopic cholecystectomy was performed. All surgeries were performed by the same surgical team with extensive experience and advanced skills in laparoscopic surgery. The timing of the intervention was determined based on the onset of symptoms, such as dyspepsia, nausea, vomiting, or abdominal pain, as reported by patients during admission. The definition of early cholecystectomy used in this study is based on the recommendations of the Tokyo Guidelines 2018 [4]. In this retrospective study, surgeries performed within seven days of symptom onset were classified as early cholecystectomy. Those who underwent surgery between one and six weeks after symptom onset were categorized as the intermediate surgery group, while patients who underwent surgery more than six weeks after symptom onset were classified as the delayed surgery group.

Patients with cholelithiasis who did not have cholecystitis, those followed up for choledocholithiasis or who underwent an endoscopic retrograde cholangiopancreatography (ERCP) procedure, patients who underwent open surgery, and patients who discontinued outpatient follow-up were excluded from the study. Patients with intraoperative bile duct stones were also excluded from the study.

QoL was assessed using the Gastrointestinal Quality of Life Index (GIQLI) at six months postoperatively. Patients were interviewed face-to-face at the sixth postoperative month, and the GIQLI questionnaire was completed by the patients. The GIQLI is a 36-item questionnaire divided into five domains. It assesses gastrointestinal symptoms, physical, psychological, social functioning and disease-specific conditions. For each parameter, the answers were divided into five levels and scored using a Likert scale (0–4; 0: worst and 4: best). The total GIQLI score is 144, with higher scores indicating better QoL (Table 1) [7]. Patients were evaluated on the basis of age, gender, timing of the operation, severity of cholecystitis according to the cholecystitis severity index and GIQLI score at the sixth postoperative month. Tokyo 2018 guidelines were used to determine the severity index of acute cholecystitis [4]. Data were reported according to the STROBE Statement checklist.

**Table 1:** The gastrointestinal quality of life ındex (GIQLI)

1. How often during the past 2 weeks have you had pain in the abdomen? All of the time, most of the time, some of the time, a little of the time, never
2. How often during the past 2 weeks have you had a feeling of fullness in the upper abdomen? All of the time, most of the time, some of the time, a little of the time, never
3. How often during the past 2 weeks have you had bloating (sensation of too much gas in the abdomen)? All of the time, most of the time, some of the time, a little of the time, never
4. How often during the past 2 weeks have you been troubled by excessive passage of gas through the anus? All of the time, most of the time, some of the time, a little of the time, never
5. How often during the past 2 weeks have you been troubled by strong burping or belching? All of the time, most of the time, some of the time, a little of the time, never
6. How often during the past 2 weeks have you been troubled by gurgling noises from the abdomen? All of the time, most of the time, some of the time, a little of the time, never
7. How often during the past 2 weeks have you been troubled by frequent bowel movements? All of the time, most of the time, some of the time, a little of the time, never
8. How often during the past 2 weeks have you found eating to be a pleasure? All of the time, most of the time, some of the time, a little of the time, never
9. Because of your illness, to what extent have you restricted the kinds of food you eat? Very much, much, somewhat, a little, not at all
10. During the past 2 weeks, how well have you been able to cope with everyday stresses? Extremely poorly, poorly moderately, well, extremely well
11. How often during the past 2 weeks have you been sad about being ill? All of the time, most of the time, some of the time, a little of the time, never
12. How often during the past 2 weeks have you been nervous or anxious about your illness? All of the time, most of the time, some of the time, a little of the time, never
13. How often during the past 2 weeks have you been happy with life in general? Never, a little of the time, some of the time, most of the time, all of the time
14. How often during the past 2 weeks have you been frustrated about your illness? All of the time, most of the time, some of the time, a little of the time, never
15. How often during the past 2 weeks have you been tired or fatigued? All of the time, most of the time, some of the time, a little of the time, never
16. How often during the past 2 weeks have you felt unwell? All of the time, most of the time, some of the time, a little of the time, never
17. Over the past week, have you woken up in the night? Every night, 5–6 nights, 3–4 nights, 1–2 nights, never
18. Since becoming ill, have you been troubled by changes in your appearance? A great deal, a moderate amount, somewhat, a little bit, not at all
19. Because of your illness, how much physical strength have you lost? A great deal, a moderate amount, some, a little bit, none
20. Because of your illness, to what extent have you lost your endurance? A great deal, a moderate amount, somewhat, a little bit, not at all
21. Because of your illness, to what extent do you feel unfit? Extremely unfit, moderately unfit, somewhat unfit, a little unfit, fit
22. During the past 2 weeks, how often have you been able to complete your normal daily activities (school, work, household)? All of the time, most of the time, some of the time, a little of the time, never
23. During the past 2 weeks, how often have you been able to take part in your usual patterns of leisure or recreational activities? All of the time, most of the time, some of the time, a little of the time, never
24. During the past 2 weeks, how much have you been troubled by the medical treatment of your illness? Very much, much, somewhat, a little, not at all
25. To what extent have your personal relations with people close to you (family or friends) worsened because of your illness? Very much, much, somewhat, a little, not at all
26. To what extent has your sexual life been impaired (harmed) because of your illness? Very much, much, somewhat, a little, not at all
27. How often during the past 2 week, have you been troubled by fluid or food coming up into your mouth (regurgitation)? All of the time, most of the time, some of the time, a little of the time, never
28. How often during the past 2 weeks have you felt uncomfortable because of your slow speed of eating? All of the time most of the time, some of the time, a little of the time, never
29. How often during the past 2 weeks have you had trouble swallowing your food? All of the time, most of the time, some of the time, a little of the time, never
30. How often during the past 2 weeks have you been troubled by urgent bowel movements? All of the time, most of the time, some of the time, a little of the time, never
31. How often during the past 2 weeks have you been troubled by diarrhea? All of the time, most of the time, some of the time, a little of the time, never
32. How often during the past 2 weeks have you been troubled by constipation? All of the time, most of the time, some of the time, a little of the time, never
33. How often during the past 2 weeks have you been troubled by nausea? All of the time, most of the time, some of the time, a little of the time, never
34. How often during the past 2 weeks have you been troubled by blood in the stool? All of the time, most of the time, some of the time, a little of the time, never
35. How often during the past 2 weeks have you been troubled by heartburn? All of the time, most of the time, some of the time, a little of the time, never
36. How often during the past 2 weeks have you been troubled by uncontrolled stools? All of the time, most of the time, some of the time, a little of the time, never

Calculation of the score: Most desirable option: 4 points; Least desirable option: 0 points; GIQLI score: sum of the points

## Statistical analysis

The data obtained from this study were summarized using descriptive statistics. Continuous variables (numerical) were presented as mean ± standard deviation or median and minimum and maximum values depending on their distribution. Categorical variables were expressed as numbers and percentages. The normality of the numerical variables was evaluated using the Shapiro–Wilk, Kolmogorov–Smirnov, and Anderson–Darling tests.

The Mann–Whitney U test was used to compare non-normally distributed numerical variables between two independent groups. The Kruskal–Wallis H test was applied to evaluate overall differences among three or more independent groups. If the Kruskal–Wallis H test indicated a significant difference, the Dwass-Steel-Critchlow-Fligner test was used for pairwise comparisons between groups.

To assess the relationship between non-normally distributed numerical variables such as age and GIQLI scores, Spearman’s Rho correlation coefficient was used.

Linear regression analysis was conducted to predict GIQLI scores based on varying times and types of surgery, Tokyo Index scores, gender, and age. Univariate linear regression models were used to measure the independent effect of each variable and multivariate linear regression models to measure the effect of each variable when all other variables were controlled. Beta coefficients, 95% confidence intervals (CIs), and P-values were used to evaluate the effects of variables on GIQLI scores.

Statistical analyses were performed with Jamovi (Version 2.3.28) and JASP (Version 0.17.3) software programs and the level of statistical significance was set at p = 0.05.

## Results

The mean age of a total of 201 study participants including 132 female (65.7%), and 69 male (34.3%) patients who underwent laparoscopic cholecystectomy, was 56.0 ± 14.9 years. According to the time of surgery, the number of patients who underwent early (30.8%; n = 62), intermediate (16.9%; n = 34), and delayed (52.2%; n = 105) cholecystectomy was recorded. The median GIQLI score was 116. According to the Tokyo index, the severity of cholecystitis was mild in 62.2% (n = 125), moderate in 29.9% (n = 60), and severe in 8% (n = 16) of the patients (Table 2). A significant correlation was not detected between patient age and GIQLI scores (p = 0.214) (Table 2).

**Table 2:** Demographic and clinical data of the patients who underwent cholecystectomy

		Total (n = 201)
Age (Mean ± SD)		56.0 ± 14.9
Gender (n (%))	Female	132 (65.7)
	Male	69 (34.3)
Time of surgery (n (%))	Early cholecystectomy	62 (30.8)
	Intermediate cholecystectomy	34 (16.9)
	Delayed cholecystectomy	105 (52.2)
GIQLI score (Median [Min.–Max.])		116.0 [96.0 – 134.0]
Tokyo indeks (n (%))	Mild	125 (62.2)
	Moderate	60 (29.9)
	Severe	16 (8.0)

*SD* standard deviation

Of the 125 patients with a mild Tokyo severity index, 44 (35.2%) underwent early cholecystectomy, 19 (15.2%) had intermediate cholecystectomy, and 62 (49.6%) underwent delayed cholecystectomy. Meanwhile, according to the Tokyo severity index, among 60 moderate acute cholecystitis cases, 16 (26.7%) underwent early cholecystectomy (EC), 11 (18.3%) had intermediate cholecystectomy (IC), and 33 (55%) underwent delayed cholecystectomy (DC). Among the 16 patients with severe cholecystitis, early cholecystectomy was performed on 2 (12.5%) patients, intermediate cholecystectomy on 4 (25%) patients, and delayed cholecystectomy on 10 (62.5%) patients.

According to univariate analysis, GIQLI scores did not show a significant difference according to gender and severity of cholecystitis (Tokyo index scores) (p = 0.439 and p = 0.056, respectively). According to the time of surgery, GIQLI scores were significantly lower in cholecystectomies performed in the intermediate group compared to both early and delayed cholecystectomies (p < 0.001 and p = 0.010, respectively). GIQLI scores were similar in early and delayed cholecystectomies (p = 0.199) (Table 3).

**Table 3:** Comparison of GIQLI scores in cholecystectomy patients according to gender, timing of surgery, and severity of cholecystitis

		Median GIQLI score [Min. − Max.]	p-value
Gender	Female (n = 132)	117.0 [96.0 – 134.0]	0.439**
	Male (n = 69)	115.0 [96.0 – 132.0]	
Time of surgery	Early cholecyctectomy (n = 62)	121.0 [96.0 – 131.0]	0.001*
	Intermediate cholecystectomy (n = 34)	110.5 [98.0 – 128.0]	
	Delayed cholecystectomy (n = 105)	116.0 [96.0 – 134.0]	
Disease severity (Tokyo Index-18)	Mild (n = 125)	117.0 [96.0 – 132.0]	0.056*
	Moderate (n = 60)	115.5 [96.0 – 131.0]	
	Severe (n = 16)	111.0 [96.0 – 134.0]	

^*^Kruskal–Wallis H test, **Mann–Whitney U test

According to univariate analysis, the time of surgery had a significant effect on GIQLI scores, while Tokyo index, age, and gender had no significant effect on GIQLI scores (p > 0.05 for each). Accordingly, GIQLI scores decreased by 6.63 units in cholecystectomies performed during the intermediate period compared to early cholecystectomy operations (p < 0.001), while GIQLI scores did not change significantly in delayed cholecystectomies (p = 0.174).

Similarly, in multivariate analyses, GIQLI scores decreased by 5.5 units (p < 0.001) in the intermediate cholecystectomy group compared to early cholecystectomy group, and no effect of delayed cholecystectomy group was found (p = 0.155). In addition, GIQLI scores decreased by 5.53 units (p = 0.006) in severe patients when mild cholecystitis patients were taken as reference according to the Tokyo index (Table 4).

**Table 4:** Factors affecting gastrointestinal QoL in cholecystectomized patients

Linear regression model predicting GIQLI scores	Univariate linear regression		Multivariate linear regression	
	Beta [95% CI]	p-value	Beta [95% CI]	p-value
Time of surgery: ref. = Early				
Intermediate cholecystectomy	− 6.63 [-10.03 – -3.24]	< 0.001	− 5.50 [− 8.72 – − 2.28]	< 0.001
Delayed cholecystectomy	− 1.77 [-4.32 – 0.77]	0.174	− 1.71 [− 4.05 – 0.64]	0.155
Tokyo index: ref. = Mild				
Moderate	− 1.72 [-0.83 – 4.27]	0.186	− 0.87 [− 1.47 – 3.21]	0.469
Severe	− 3.97 [-8.28 – 0.34]	0.073	− 5.53 [− 9.45 – − 1.60]	0.006
Gender: Male vs. Female	− 0.72 [-3.16 – 1.72]	0.564		
Age	− 0.05 [-0.12 – 0.03]	0.242		

Table 4 presents the results of the linear regression analysis predicting the GIQLI scores. Both univariate and multivariate regression analyses are included. Effects of variables on GIQLI scores are assessed using beta confidences, 95% confidence intervals (CI), and p-values. A total of 201 observations were analyzed. The R-squared value is 0.2412, and the Akaike Information Criterion (AIC) value is calculated as 1387.98. These values indicate the model’s fit to the data and the overall quality of the analysis

When the groups were compared based on the timing of cholecystectomy in terms of operation duration, intraoperative blood loss, and postoperative hospital stay, no statistically significant differences were observed (p > 0.05) (Table 5).

**Table 5:** Comparison of ıntraoperative and postoperative data based on the timing of cholecystectomy

	Early cholecystectomy (n = 62) [Min. – Max.]	Intermediate cholecystectomy (n = 34) [Min. – Max.]	Delayed cholecystectomy (n = 105) [Min. – Max.]	p-value
Operation time (minutes)	98 [29–228]	108 [44–292]	101 [32–256]	0.355*
Blood loss (ml)	95 [10–490]	115 [10–570]	105 [10–540]	0.141*
Postoperative discharge (days)	3.11 [1–17]	3.83 [1–27]	3.25 [1–22]	0.211*

^*^Kruskal–Wallis H test

A total of three cases of postoperative jaundice were observed (1 EC, 1 IC, 1 DC). Bile leakage occurred in six cases (1 EC, 2 IC, 3 DC). Surgical site infection was observed in six cases (1 EC, 2 IC, 3 DC). Intra-abdominal collection was noted in five cases (1 EC, 2 IC, 2 DC). Atelectasis was observed in a total of three cases, with one case in each group (1 EC, 1 IC, 1 DC). Atrial fibrillation was observed in a total of four cases (1 EC, 1 IC, 2 DC). Patients with jaundice underwent magnetic resonance cholangiopancreatography (MRCP) followed by successful ERCP. The six patients with surgical site infections were treated with oral antibiotics. Among the six patients with bile leakage, four were managed conservatively due to low-volume leakage that resolved spontaneously. The remaining two patients required ERCP-guided stent placement and were closely monitored. Of the patients with intra-abdominal collections, four were treated successfully with oral antibiotics, while one required the placement of an intra-abdominal catheter. Patients with pulmonary and cardiac complications were treated appropriately and discharged without further issues. No mortality occurred in any of the cases (Table 6).

**Table 6:** Comparison of postoperative complications based on the timing of cholecystectomy

Complications	Early cholecystectomy (n = 62)	Intermediate cholecystectomy (n = 34)	Delayed cholecystectomy (n = 105)
Postoperative jaundice	1 (1.6%)	1 (2.9%)	1 (1.0%)
Bile leakage	1 (1.6%)	2 (5.9%)	3 (2.9%)
Surgical site infection	1 (1.6%)	2 (5.9%)	3 (2.9%)
Intra-abdominal collection	1 (1.6%)	2 (5.9%)	2 (1.9%)
Atelectasis	1 (1.6%)	1 (2.9%)	1 (1.0%)
Atrial fibrillation	1 (1.6%)	1 (2.9%)	2 (1.9%)

## Discussion

As is proven in some studies, laparoscopic surgery significantly improves QoL in patients with symptomatic cholelithiasis [8, 9]. With the use of laparoscopy in the surgical field, studies evaluating QoL have mostly focused on early and delayed laparoscopic cholecystectomy. There is limited information on the effect of cholecystectomies performed in the intermediate period on the QoL of patients.

In this study, we evaluated the effects of the timing of laparoscopic cholecystectomies performed for acute cholecystitis on postoperative QoL of patients. In addition, we aimed to contribute to the literature on the effects of intermediate laparoscopic surgeries on QoL, which has been studied at a lesser rate in the literature. Our findings have shown that early term surgery provides a significant improvement in QoL. In particular, the QoL scores of patients who underwent early cholecystectomy were found to be significantly higher than those in the intermediate group. Again, the mean GIQLI scores of the early cholecystectomized patients were higher than those who underwent delayed surgery, despite the lack of any statistically significant intergroup difference. These findings are consistent with the recommendations in the current literature that early surgery reduces the incidence of wound site infections and shortens the length of hospital stay [3, 6].

In a study comparing early and delayed laparoscopic cholecystectomies performed for acute cholecystitis, cholecystectomies performed in the first 72 h after the onset of symptoms were defined as early, and those performed after 6 − 8 weeks as delayed cholecystectomies. In the study, a higher rate of patient satisfaction was reported by patients who underwent early surgery [10]. In a meta-analysis, XD Wu et al. compared early and delayed laparoscopic cholecystectomies in terms of cost-effectiveness and QoL and found that early laparoscopic cholecystectomies were associated with lower cost, decreased labor loss, and higher patient satisfaction and QoL [11]. Another study conducted to compare the cost-effectiveness and QoL of early and delayed laparoscopic cholecystectomies showed that early laparoscopic cholecystectomy was less costly and resulted in better QoL than delayed surgery [12]. However, in another randomized controlled study evaluating early and delayed laparoscopic cholecystectomies, comparable rates of cost-effectiveness, but higher health-related QoL scores were detected in the delayed laparoscopic cholecystectomy group [13].

Johansson et al. found a significant decrease in gastrointestinal symptoms and an increase in QoL at the first postoperative month in patients who underwent early laparoscopic cholecystectomies compared to those who underwent delayed laparoscopic cholecystectomies, but no significant difference was found between the groups at the sixth postoperative month [14].

One of the most significant studies on the timing of acute cholecystectomy, the AC/DC trial, compared early cholecystectomy performed within the first 24 h after the diagnosis of acute cholecystitis with delayed cholecystectomy performed between 7 and 45 days later. The study demonstrated that early surgery was superior in reducing morbidity and lowering medical expenses [15]. However, while the AC/DC trial primarily compared early and delayed cholecystectomy in terms of mortality, morbidity, and complications, our study specifically evaluates the impact of early, intermediate, and late cholecystectomy on quality of life. Similar to the AC/DC trial, our study found significantly higher GIQLI scores in the early cholecystectomy group compared to the intermediate group, indicating better outcomes.

Conversely, another study comparing 74 patients who underwent surgery within the first 7 days with 42 patients who underwent surgery after day seven found no significant difference in mortality rates or postoperative hospital stays between the two surgical timings [16]. When assessing patient outcomes based on the timing of cholecystectomy, it is crucial to consider that intermediate cholecystectomy is generally performed out of necessity rather than preference. Intermediate cholecystectomy is typically conducted in patients who did not seek medical attention during the early phase, experienced worsening symptoms, or developed complications during follow-up. Therefore, the lower GIQLI scores observed in the intermediate cholecystectomy group should not be interpreted as an indication that these patients would have had better outcomes if they had not undergone surgery during this period. On the contrary, the lower GIQLI scores in the intermediate group compared to the early and late groups reflect the necessity of surgical intervention and the clinical conditions under which the surgery was performed.

Patients in the intermediate group are often managed with conservative approaches such as antibiotic therapy or drainage instead of emergency surgery during an acute cholecystitis episode. In this period, factors such as prolonged infection, persistent inflammation, and worsening symptoms may contribute to the lower QoL scores observed in the intermediate group, as seen in our study. Additionally, fibrosis and adhesion formation can complicate the surgical procedure, leading to longer operative times, increased blood loss, and higher rates of postoperative complications [5, 15, 16]. Although not statistically significant, our study observed longer operative times, greater mean blood loss, and prolonged postoperative discharge durations in the intermediate cholecystectomy group compared to the other groups. Furthermore, complications such as bile leakage, wound infections, and postoperative intra-abdominal collections were more frequently observed in the intermediate cholecystectomy group.

In our study, QoL reported by patients differed significantly between patient groups in terms of the severity of cholecystitis as evaluated using the Tokyo Index. The decrease in QoL in severe cholecystitis cases indicates that these patients experience more severe symptoms and complications. This finding suggests that the severity of cholecystitis is an important factor affecting postoperative QoL. The distribution of mild, moderate, and severe cases across the early, intermediate, and delayed groups may influence post-operative QoL outcomes. However, in our study multivariate regression analysis demonstrates that intermediate cholecystectomy negatively affects QoL independently of disease severity. Severe cases also show an independent negative impact on QoL, emphasizing the importance of both surgical timing and disease severity in determining outcomes. Some studies have shown that the complication rates and the possibility of conversion to open surgery in patients with moderate and severe cholecystitis according to the Tokyo index are higher than those with mild cholecystitis [17, 18].

In the present study, factors such as gender and age did not have a significant effect on GIQLI scores. As a result of advances in the field of medicine, life expectancy has increased, with resultant increase in the incidence of geriatric diseases. One of the most common surgical diseases encountered in individuals in the geriatric age group is cholelithiasis, and the need for surgical intervention increases when acute cholecystitis develops in elderly patients [19]. Although we found that age had no effect on the GIQLI scores, some studies in the literature have reported that elderly patients who underwent laparoscopic cholecystectomy had a longer postoperative hospital stay and transition to oral nutrition, a higher complication rate, and a more frequent switch to open surgery [20, 21]. Although the effect of gender on the GIQLI scores of laparoscopic cholecystectomy patients has not been adequately investigated in the literature, it has been reported that laparoscopic cholecystectomy is more difficult to perform and time-consuming in men than in women [22].

In conclusion, our study has shown that early cholecystectomy operations provide a significant improvement in QoL of patients and reduce the risk of postoperative complications. The negative effects of intermediate surgeries on QoL emphasize how critical the timing of surgical intervention is in such cases. Future studies should further investigate the long-term outcomes of early surgery and its effects in different patient groups.

The retrospective design, relatively small sample size, and short-term postoperative monitoring (only 6 months) are the limiting factors of our study. In addition, since we only operated on emergency cases in the intermediate period and excluded patients whose postoperative health conditions were stable, we could not fully evaluate the effects of complications on QoLs of patients.

## Conclusion

Our study shows the positive effects of early intervention in the treatment of acute cholecystitis on QoL. These findings, consistent with existing literature, highlight the importance of early intervention. In general, studies measuring QoL after cholecystectomies performed for acute cholecystitis focus on the early and late periods and often ignore the intermediate period. We believe that our study will contribute to the literature in this regard. Furthermore, we believe that studies involving larger patient populations should be conducted to better understand the effects of surgical timing on QoL in cholecystitis patients. Future research could also explore the impact of early intervention on patients with diverse demographic characteristics in greater detail, enabling the development of more specific guidelines in this field.

## Supplementary Information

Below is the link to the electronic supplementary material.

Supplementary file1 (DOCX 41 KB)
