# Correction: Impact of surgical timing on postoperative quality of life in acute cholecystitis: a comparative analysis of early, intermediate, and delayed laparoscopic cholecystectomy

**DOI:** 10.1007/s00464-025-11648-x

**Published:** 2025-03-04

**Authors:** Azad Gazi Şahin, Erman Alçı

**Affiliations:** Department of General Surgery, School of Medicine, Balikesir University Hospital, 10463 Balikesir, Turkey

**Correction to: Surgical Endoscopy** 10.1007/s00464-025-11620-9

The original online version of this article was revised to replace placeholder text in the Materials and methods section as follows:

This is an observational, retrospective cohort study in which a total of 201 patients who underwent surgery for acute cholecystitis between May 2019 and February 2023 in the General Surgery clinics of Balikesir University Hospital were examined. The study was approved by Balikesir University Clinical Research Ethics Committee (Date: 22.11.2023; No: 2023/178).

The original article has been corrected.

